# Replicable subcortical alterations linked to neurological soft signs in schizophrenia spectrum disorders

**DOI:** 10.1017/S0033291726105078

**Published:** 2026-07-06

**Authors:** Amanda Bellanti, Sebastian Volkmer, Dilsa Cemre Akkoc Altinok, Stefan Fritze, Geva A. Brandt, Heike Tost, Urs Braun, Sofie von Känel, Anastasia Pavlidou, Stephanie Lefebvre, Andreas Meyer-Lindenberg, Sebastian Walther, Dusan Hirjak

**Affiliations:** 1 Central Institute of Mental Health: Zentralinstitut fur Seelische Gesundheit, Germany; 2 University of Bern: Universitat Bern, Switzerland; 3 University of Wurzburg: Julius-Maximilians-Universitat Wurzburg, Germany; 4 Department of Psychiatry and Psychotherapy, Central Institute of Mental Health: Zentralinstitut fur Seelische Gesundheit, Mannheim, Germany

**Keywords:** basal ganglia, FSL-FIRST, MRI, neurological soft signs, schizophrenia spectrum disorders, thalamus

## Abstract

**Background:**

Neurological soft signs (NSS) are frequent in schizophrenia spectrum disorders (SSD) and have been linked to structural alterations in basal ganglia-thalamic (BGT) regions. We hypothesized that SSD patients would show BGT volume differences compared to healthy controls (HC) and that NSS severity would relate to BGT volume and surface morphology in a replicable pattern.

**Methods:**

Structural 3T T1-weighted MRI scans were obtained from 327 SSD patients and 134 matched HC in Mannheim (Germany) and Bern (Switzerland). NSS were assessed using the Heidelberg Scale and the Neurological Evaluation Scale (NES). BGT volumes were segmented using FSL-FIRST and compared across groups using general linear models adjusted for age, sex, intracranial volume, and daily antipsychotic medication. Associations with NSS scores were tested using regression analyses.

**Results:**

High-NSS compared to low-NSS SSD patients showed reduced left accumbens volume in both cohorts, with a significant main effect in the Mannheim cohort (β = −43.73, p = .002 uncorrected, p = .019 corrected) and a partial replication in the Bern cohort (β = −53.06, uncorrected p = .03, p > .05, corrected). In contrast, IF-related effects on left accumbens and bilateral thalamic volumes were cohort specific. Daily antipsychotic medication and illness duration did not mediate or moderate these associations.

**Conclusions:**

This bicentric MRI study provides converging evidence that NSS severity in SSD is associated with BGT alterations, particularly reduced left nucleus accumbens volume. However, thalamic and surface-level findings were cohort specific, indicating partial rather than uniform reproducibility. Associations were not explained by daily dosage of antipsychotic medication or illness duration.

## Introduction

Beyond prominent sensorimotor and psychomotor syndromes, such as catatonia, akathisia, and parkinsonism, individuals with schizophrenia spectrum disorders (SSD) frequently display more subtle sensorimotor and psychomotor abnormalities (Hirjak et al., 2025). These so-called neurological soft signs (NSS) refer to minor motor and sensory deficits observable during clinical examination and include impairments in coordination, sequencing of movements, and sensory integration (Fritze et al., 2020; Herold, Hirjak, & Schröder, 2018; Hirjak, Wolf, Schröder, & Thomann, 2012, 2013; Hirjak, Wolf, & Thomann, 2015). NSS are highly prevalent in SSD, affecting approximately 60%–70% of patients (Hirjak et al., 2018; Hirjak, Kubera, Thomann, & Wolf, 2018; Walther & Strik, 2012). Interestingly, NSS severity at baseline showed stability over time in a 21-year follow-up study with first-episode patients and was a significant independent predictor of negative and positive symptoms, poor functioning, and less personal recovery at follow-up (Peralta, Sánchez-Torres, & Cuesta, 2024; Pieters, Nadesalingam, Walther, & van Harten, 2022), suggesting a possible trait-feature of neuromotor dysfunction in SSD. Additionally, the presence of NSS in high-risk individuals (Dean, Bernard, Orr, & Mittal, 2014; Mittal, Dean, & Millman, 2014), antipsychotic-naïve patients (Peralta, Campos, & Cuesta, 2010), and even unaffected relatives (Niethammer, Weisbrod, & Sauer, 2000; Schappi, Stegmayer, Viher, & Walther, 2018) suggests that these signs may reflect neurodevelopmental and illness-intrinsic processes, rather than being secondary to medication effects. Their severity has been shown to increase with age in healthy controls (HC) and even more markedly SSD (Herold, Hirjak, & Schröder, 2018), highlighting a susceptibility for both neurodevelopmental and neurodegenerative processes (Volkmer et al., 2026).

Structural and functional MRI research has progressively elucidated the neurobiological underpinnings of NSS in SSD. Among the implicated substrates, the basal ganglia and thalamic regions (BGT) have emerged as critical hubs due to their integrative role in motor execution, cognitive control, and the pathophysiology of a broad range of neuropsychiatric motor phenomena, including stereotypies, tics, and perseverative behaviors (C. L. Burton et al., 2024; Vicente, Martins, & Costa, 2020). Converging evidence from multiple imaging studies has linked NSS to subcortical region-specific alterations within the caudate nucleus (Cuesta, Moreno-Izco, López-Ilundain, & Peralta, 2021; Dazzan et al., 2004; Hirjak, Wolf, Schröder, & Thomann, 2012); Janssen, Diaz-Caneja, Reig, and Arango (2009)); (Thomann, Bachmann, & Schröder, 2009), putamen (Dazzan et al., 2004; Kasparek, Prikryl, & Vanicek, 2009; Venkatasubramanian, Jayakumar, Gangadhar, & Keshavan, 2008), globus pallidus (Dazzan et al., 2004; Hirjak, Wolf, Schröder, & Thomann, 2012; Kasparek, Prikryl, & Vanicek, 2009; Venkatasubramanian, Jayakumar, Gangadhar, & Keshavan, 2008), thalamus (Dazzan et al., 2004; Janssen, Diaz-Caneja, Reig, & Arango, 2009; Thomann, Bachmann, & Schröder, 2009), as well as infratentorial regions such as cerebellum (Bottmer et al., 2005; Hirjak, Wolf, & Thomann, 2015; Mouchet-Mages & Meder, 2007; Thomann, Roebel, & Schroder, 2009; Venkatasubramanian, Jayakumar, Gangadhar, & Keshavan, 2008), and brainstem (Hirjak, Wolf, Schröder, & Thomann, 2013), respectively. Recent task-based fMRI evidence extends these structural findings by showing that BGT abnormalities in SSD are expressed dynamically across movement phases. Rashid et al. (2025a) reported reduced preparatory BOLD activation during active hand movement in regions including the putamen and thalamus. Rashid et al. (2025b) further found altered BOLD response timing during active relative to passive movement in the caudate, putamen/insula, and thalamus. These findings suggest that BGT alterations in SSD may reflect not only morphological abnormalities but also impaired neural response amplitude and timing during movement preparation and execution.

These findings align with established neurobiological models from movement disorder research, underscoring the contribution of distributed subcortical-cerebellar circuits to the pathophysiology of NSS in SSD. However, the reproducibility and generalizability of these associations remain uncertain. To date, no study has systematically examined NSS and their neural correlates across two independent cohorts, a crucial step for robust replication in SSD research.

Therefore, to investigate the relationship between NSS and BGT structure in SSD, we conducted a structural MRI study across two independent cohorts of SSD patients and two independent cohorts of HC. High-resolution MRI data were processed using automated segmentation with FSL-FIRST (Patenaude, Smith, & Jenkinson, 2011). First, we stratified SSD patients in the Mannheim (Germany) and Bern (Switzerland) cohorts into low and high NSS groups using a median split of the NSS total scores. This enabled us to examine NSS-specific differences in BGT morphology within SSD. Second, we tested whether the identified BGT volume differences correlated with NSS severity across the full range of scores. Third, we compared low- and high-NSS SSD groups to age- and sex-matched HC to clarify whether NSS-related BGT alterations reflected SSD-associated volume variation or true volume reductions. Finally, we investigated whether the association between BGT alterations and NSS was mediated or moderated by daily antipsychotic medication exposure or duration of illness (DOI).

## Methods

### Study participants

In this study, we examined two independent cohorts of SSD patients and two independent cohorts of HC from different studies conducted at the Central Institute of Mental Health (CIMH) in Mannheim, Germany and the in- and outpatient clinics at the University Hospital of Psychiatry and Psychotherapy in Bern (UHPB), Switzerland.

The Mannheim SSD cohort (whiteCAT and NSS studies at CIMH) (Hirjak et al., 2024) comprised 209 individuals aged 18–65 with a diagnosis of SSD according to ICD-10 criteria; the German Mini Diagnostic Interview for Mental Disorders (Mini-DIPS; Margraf, 1994) was administered to systematically assess and exclude relevant psychiatric comorbidities. The study participants in the whiteCAT cohort had been recruited between April 2022 and March 2025 and used in previous studies of our group (Altinok et al., 2025; Fritze, Brandt, Meyer-Lindenberg, & Hirjak, 2025; Peretzke, Neher, & Hirjak, 2025). The study participants from the NSS cohort had been recruited between February 2017 and October 2018 and have been used in previous studies of our group as well (Fritze et al., 2022; Northoff et al., 2022; Sambataro et al., 2021). For both samples, diagnoses were made by staff psychiatrists and confirmed using the examination of the case notes (GAB, DCAA, SF, and DH). All relevant study procedures (e.g. psychopathological rating scales, neuropsychological assessments, and sensorimotor assessments as well as MRI scan) were completed within 7 days.

*Mannheim HC cohort (URBN and BMBF studies at CIMH)* (Fritze et al., 2022; Geiger et al., 2018) consists of 75 healthy controls aged 18–58 years stemming from the BMBF study and the URBN study recruited onsite at CIMH in Mannheim, Germany. Healthy controls were selected from an initial sample of 333 individuals to match the patient group in age and sex using propensity score matching (nearest neighbor matching on Mahalanobis distance). Matching was performed with replacement to ensure that each patient could be matched to a close control. The MatchIt R-Package Version 4.7.2 (2025-05-30) was used for this procedure. For subsequent analyses, only unique controls were retained to preserve independence of observations.

*Bern SSD and HC cohorts* (subsamples from the OCoPS and BrAGG-SoS trials) consisted of 118 SSD patients and 59 HC. These participants have been recruited from the in- and outpatient clinics at the University Hospital of Psychiatry and Psychotherapy in Bern, Switzerland, and used in previous studies (Walther et al., 2024; Walther et al., 2025). Diagnoses were given according to DSM-5, following structured interviews and review of case files by trained psychiatrists.

Patients in both cohorts were excluded if they: (i) were aged <18 or >65 years; (ii) had a history of brain trauma or neurological disease (especially primary movement disorders); (iii) had alcohol/substance use disorder within 12 months prior to participation; or (iv) had MRI contraindications. The local Ethics Committees I and II (Medical Faculty Heidelberg and Medical Faculty Mannheim at Heidelberg University, Germany) and the local ethics committee in Bern, Switzerland, approved the studies. We obtained written informed consent from all study participants after all aims and procedures of the study had been fully explained and before starting assessments.

### Clinical assessment

SSD patients in both cohorts were examined during in- or outpatient treatment, typically after partial remission of acute psychopathological symptoms.

In the Mannheim SSD cohort, the Heidelberg NSS scale was used to investigate neurological soft signs (Schröder et al., 1991). The Heidelberg NSS scale comprises 16 items grouped into five subdomains reflecting distinct aspects of neurological functioning. Motor coordination (MOCO) includes Ozeretzki’s test, diadochokinesia, pronation/supination, finger-to-thumb opposition, and speech articulation, capturing fine motor control, rhythm, and coordination of sequential movements. Integrative functions (IF) assess basic sensorimotor integration and postural control through station and gait, tandem walking, and two-point discrimination. Complex motor tasks (COMT) include the finger-to-nose and fist-edge-palm tests, indexing higher-order motor sequencing and coordination. Right/left and spatial orientation (RLSPO) comprises right–left orientation, graphesthesia, face-hand test, and stereognosis, reflecting spatial processing and sensory integration abilities. Hard signs (HS), including the arm holding test and mirror movements, capture more pronounced or ‘non-soft’ neurological abnormalities. Each item is rated on a 4-point scale from 0 (no abnormality) to 3 (marked or consistently present abnormality) based on standardized examination procedures. Subscale scores are calculated by summing the respective items, and a total NSS score is derived as the sum across all domains. The original German manual of the Heidelberg NSS scale can be obtained from the corresponding authors upon reasonable request. Access to derived data and analysis scripts is also available upon reasonable request, subject to the signing of an appropriate data transfer agreement and in accordance with applicable ethical and data protection regulations. A sufficient internal reliability and test–retest reliability have been established previously (Bachmann, Bottmer, & Schröder, 2005; Schröder et al., 1991). For psychopathological assessments, patients were examined with the Positive and Negative Syndrome Scale (PANSS; including the positive, negative, and general subscores) (Kay, Fiszbein, & Opler, 1987). Cognitive assessment was performed with the Brief Cognitive Assessment Tool for Schizophrenia (B-CATS) (Hurford et al., 2011), including Trail Making Test B (TMT-B), Digit Symbol Substitution Test (DSST), and Category Fluency (CF). Global functioning was investigated with the Global Assessment of Functioning (GAF) (Hall, 1995) scale.

In the Bern SSD cohort, the Neurological Evaluation Scale (NES), which covers similar items as the Heidelberg NSS scale (e.g. Ozeretski’s test, finger-thumb opposition, and tandem walk) was applied (Buchanan & Heinrichs, 1989) to assess NSS. This battery consists of 26 items in total, with 5 items assessing sensory integration, 4 items assessing motor coordination, 4 items assessing sequencing of complex motor tasks, and the remaining 13 items assessing other aspects of neurological soft signs like mirror movements, synkinesis, tremor, and Romberg test. For psychopathological assessments, patients were examined with the PANSS (Kay, Fiszbein, & Opler, 1987). Global functioning was investigated with the GAF (Hall, 1995) scale. The current daily doses of antipsychotics was converted to olanzapine equivalents (OLZ) (Leucht et al., 2015) in both cohorts. For inpatients, medication intake was supervised by clinical staff, whereas adherence could not be systematically verified in patients treated in outpatient settings.

### Structural MRI data acquisition

Mannheim SSD and HC cohorts (*whiteCAT/NSS and URBN/BMBF studies*): for the whiteCAT SSD cohort and for the URBN cohort, MRI scans were acquired at the Central Institute of Mental Health, Mannheim, Germany, using a 64-head coil 3.0 Tesla Siemens Magnetom whole-body imaging system and a T1-weighted magnetization-prepared rapid gradient-echo (MP-RAGE) sequence with the following parameters: repetition time (ms): 2000; echo time (ms): 3.03; inversion time (ms): 900; flip angle: 9°; number of averages: 1; slice thickness (mm): 1; image columns: 256; image rows: 256; phase encoding direction: ROW; voxel size *x* (mm): 1; voxel size *y* (mm): 1; number of volumes: 1; number of slices: 192; number of files: 192.

For the NSS and BMBF cohorts, MRI scans were acquired at the Central Institute of Mental Health, Mannheim, Germany, using a 3.0 Tesla Siemens Trio whole-body imaging system and a T1-weigthed magnetization-prepared rapid gradient-echo (MP-RAGE) sequence with the following parameters: repetition time (ms): 2530; echo time (ms): 3.8; inversion time (ms): 1100; flip angle: 7°; number of averages: 1; slice thickness (mm): 1; image columns: 256; image rows: 256; phase encoding direction: ROW; voxel size x (mm): 1; voxel size y (mm): 1; number of volumes: 1; number of slices: 176; number of files: 176.

*Bern SSD and HC cohorts:* MRI images were acquired using two 3-Tesla scanners, a Siemens MAGNETOM Prisma and a Siemens MAGNETOM Tim Trio (Siemens Healthineers, Erlangen, Germany). Both systems were equipped with a 20-channel radiofrequency head coil and located at the University Hospital of Bern, Switzerland. The same pulse sequences and acquisition parameters were used on both scanners. A 3D T1-weighted magnetization-prepared rapid acquisition gradient echo (MP2RAGE) sequence was employed, yielding 176 sagittal slices with a 240 × 256 matrix and a field of view of 240 × 256 mm^2^, resulting in an isotropic voxel size of 1 mm^3^. Additional parameters for the anatomical sequence included a repetition time (TR) of 5000 ms, echo time (TE) of 2.98 ms, and inversion times (TI1/TI2) of 700 ms and 2500 ms, respectively, with flip angles of 4° and 5° for the two readouts. Parallel imaging was performed using Siemens’ GRAPPA (generalized autocalibrating partially parallel acquisition) technique with an acceleration factor of 3 in the phase-encoding direction. The total scan duration was 8 minutes and 22 seconds.

### Subcortical volumes and shape computation

All structural MRI images underwent the FSL fsl_anat structural pipeline (FMRIB Software Library version 6.0.6) (Patenaude, Smith, & Jenkinson, 2011). This comprises among others brain extraction with the BET tool (Smith, 2002), FAST (Zhang, Brady, & Smith, 2001) for tissue-type segmentation and FIRST (Patenaude, Smith, & Jenkinson, 2011) for subcortical structure segmentation. Registration to the Montreal Neurological Institute (MNI152) standard space at 1 mm isotropic resolution (CIT) as well as segmentation outputs underwent quality check prior inclusion in statistical analysis. Subcortical volumes were computed out of the boundary-corrected segmentation outputs of the FIRST tool. Total intracranial volume (ICV) was calculated as the sum of white and gray matter out of partial volume estimation images calculated from the FAST tool. For vertex-wise shape analysis, the bvars files (binary shape parameter files) generated by FIRST were used as input for surface-based statistical analysis. These files encode the modeled vertex displacements from the subcortical surface templates. Using the first_utils function, the shape data were converted to a format suitable for group comparison.

### Statistical analyses

Statistical analyses were performed using R version 4.4.42 via R-Studio version 2024.12.1 (R Core Team, 2024). Within the Mannheim and Bern cohorts separately, SSD patients were stratified into low- and high-NSS groups using median splits of the total and subscale scores of the NSS and NES scales. For simplicity and readability, the term “NSS” is used hereafter to refer collectively to both measures. Median splits were applied separately within each cohort to account for cohort-specific NSS score distributions and to define low- and high-NSS groups relative to each cohort’s own distribution. This approach made the stratification comparable across cohorts in terms of relative NSS burden and allowed stratified analyses to identify potentially divergent relationships that might be masked in continuous analyses. Consistent with previous NSS studies using categorical low- versus high-NSS groupings (Dazzan et al., 2004; Ismail, Cantor-Graae, Cardenal, & McNeil, 1998), this strategy was chosen in the absence of validated fixed cut-off scores with sufficient discriminative validity (Cuesta et al., 2002). A high-score group was formed with individuals scoring >= the median, and a low-score group was formed with individuals scoring < the median for both the total score and each subscale. To assess NSS-related effects on BGT morphology, an analysis of covariance (ANCOVA) was conducted estimating between-group differences with NSS group (low vs high) as fixed factors and age, sex, medication, and ICV as covariates. In a second step, we further explored the association between BGT volumes showing significant differences and the NSS score via linear regression models with BGT volumes as predictors and NSS scores as continuous outcome within each group. Finally, for each subscale, the high-score and low-score groups were compared with a matched healthy control sample controlling for age, sex, and ICV. Matching was performed using the MatchIt package in R (Ho, Imai, King, & Stuart, 2011), applying a Nearest Neighbor algorithm with Mahalanobis distance as the proximity metric. This strategy was preferred over propensity-based methods as it satisfies the condition of Equal Percent Bias Reduction, ensuring the balance is improved across all linear combinations of the covariates simultaneously (King & Nielsen, 2019).

To examine possible moderating effect of daily antipsychotic medication and DOI on significant associations between BGT volumes and NSS/NES subscale, mediation and moderation analyses were conducted. Mediation analyses were performed using the mediation package in R (Tingley et al., 2014). For each model, we estimated the Average Causal Mediation Effect (ACME), representing the indirect effect of the predictor through the mediator, and the Average Direct Effect (ADE), representing the effect of the predictor on the outcome after accounting for the mediator. Two separate linear regression models were constructed for each analysis: (1) a mediator model regressing the mediator on the predictor and (2) an outcome model regressing the outcome on both the predictor and the mediator. Both models were adjusted for total cerebral volume, sex, and age as covariates. To account for potential non-normality in the sampling distribution, uncertainty estimates and p-values for the ACME and ADE were generated using non-parametric bootstrapping with 5,000 resamples. We employed non-parametric bootstrapping with 5000 resamples to estimate the mediation effects because it does not require the assumption of normality for the sampling distribution of the indirect effect a×b , thereby providing superior statistical power and more accurate confidence intervals than traditional asymptotic tests like the Sobel test. Moderation analyses were performed using multiple linear regression. For each analysis, a moderation effect was tested by including an interaction term (Predictor × Moderator) within the regression model. This approach allowed us to determine if the moderator significantly influenced the magnitude or direction of the relationship between the predictor and the dependent variable. All models were adjusted for relevant covariates (age, sex, medication, and ICV). Statistical significance for the moderation was determined by the p-value associated with the coefficient of the interaction term.

For BGT structures showing significant volumetrical differences between low NSS and high NSS groups, vertex-wise shape analysis was performed to explore their morphological differences as well. Permutation testing via FSL’s *randomise* tool with 10,000 permutations was used to assess statistical significance (Winkler & Nichols, 2014). A general linear model (GLM) was constructed to model group differences. A second linear model was constructed to explore the associations between NSS scores and morphology in each group. For each structure, displacement values at each surface vertex were regressed against the clinical score. Statistical non-parametric permutation testing and threshold-free cluster enhancement (TFCE) were applied to identify significant clusters of vertices. Both group differences and correlation were tested in both directions and age, sex, medication, and ICV included as covariates in every model. All continuous variables were zero-centered. Significance was assessed at p < 0.05 for corrected p-values. The TFCE-corrected p-values maps given from *randomise* were thresholded for significant values and then projected on the average surfaces to visualize significant morphological differences and associations with NSS scores. Where no significant cluster was detected, t-values maps given from *randomise* were projected on the average surfaces instead in order to visualize effects directionality and magnitude.

## Results

### Demographic and clinical characteristics

Demographic characteristics of all study cohorts are shown in Table 1. Specific clinical characteristics of the two patient cohorts are shown in Table 2.

**Table 1.** Demographic characteristics of SSD patients and healthy controls (HC) in Mannheim and Bern cohort Table 1. long description.

	Mannheim cohort		Bern cohort	
Demographics	SSD (n = 209)	HC (n = 75)	SSD (n = 118)	HC (n = 59)
Age, mean (SD)[range], years	38.14 (12.01) [18–65]	36.75 (11.65) [18–58]	36.61 (12.14) [18–60]	37.41 (12.44) [20–59]
	*p = .39*		*p = .74*	
	*Mean age patients samples: p = .29* *Mean age control samples: p = .75*			
Sex, n male (%)	124 (59%)	43 (57%)	62 (52.54%)	31 (52.54%)
	*p = .23*			
Right-handed, n (%)	93.3%	n.a.	n.a.	n.a.

**Table 2.** Clinical characteristics of SSD patients in Mannheim and Bern cohorts (n = 327) Table 2. long description.

Cohort	Mannheim cohort		Bern cohort		Group differences
Clinical characteristics	SSD (n = 209)		SSD (n = 118)		
NSS total score, mean (SD)	19.57 (8.81)^a^		n.a		n.a.
NES total score, mean (SD)	n.a		13.69 (9.37)^b^		n.a
PANSS total score, mean (SD)	66.8 (19.26)		70.69 (18.56)		*p = .08*
PANSS negative symptoms, mean (SD)	17.18 (7.42)		20.03 (6.92), na = 1		*p < .001*
PANSS positive symptoms, mean (SD)	15.53 (6.29)		14.64 (4.92), na = 1		*p = .19*
DOI, mean (SD), years	10.8 (10.94), NA = 2		10.63 (10.41)		*p = .89*
OLZ equivalent, mean (SD), mg/day	16.39 (14.88)		14.02 (11.37)		*p = .13*
NSS/NES subscales, mean (SD), median	Motor coordination	7.50 (4.15), 7	Motor coordination	2.06 (2.23), 1	
	Integrative functions	3.42 (1.91), 3	Integrative functions	2.54 (2.54), 2	
	Complex motor tasks	3.41 (2.17), 3	Sequencing	4.23 (3.34), 4	
	Spatial tasks	2.96 (2.59), 3	Others	5.11 (4.60), 4	
	Hard signs	3.09 (1.96), 3			
Currently medicated (n) Currently unmedicated (n)	n = 195 n = 14		n = 107 n = 5 na = 6		*p = .42*
SAS, mean (SD)	3.44 (2.95)		na		
AIMS, mean (SD)	1.07 (2.47)		0.92 (2.82)		*p = .617*
NCRS, mean (SD)	4.23 (4.19)		na*		
BFCRS, mean (SD)	na*		3.83 (4.14)		

*Note*: NSS, Heidelberg Neurological Soft Signs Scale; NES, Neurological Evaluation Scale; PANSS, Positive and Negative Syndrome Scale; DOI, duration of illness; OLZ, olanzapine equivalent; SD, standard deviation; SAS, Simpson Angus Scale, AIMS, Abnormal Involuntary Movements Scale; NCRS, Northoff Catatonia Rating Scale; BFCRS, Bush-Francis Catatonia Rating Scale. ^a^Heidelberg NSS scale. ^b^Neurological Evaluation Scale. *BFCRS and NCRS scores were not available for all participants in the Mannheim and Bern cohorts; therefore, descriptive statistics (mean ± SD) were not calculated or reported for these measures.

### Group differences in SSD patients (ANCOVA)

**Figure 1.**
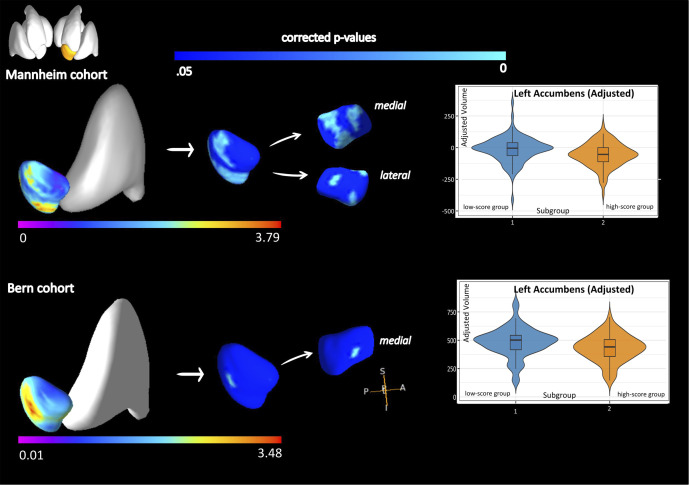
Significantly reduced left nucleus accumbens volume was observed in high-NSS (subgroup 2) compared to low-NSS patients (subgroup 1) in both cohorts (*p* < .05; corrected). Left: raw t-maps and corrected p-values maps of surface displacements in the left accumbens. Right: adjusted left accumbens volumes in the two subgroups. Figure 1. long description.

*NSS total score:* In the Mannheim cohort, the high-NSS group showed significantly reduced left accumbens volume (*β* = −43.73, *p* = .002 uncorrected, *p* = .019 corrected), which was partialy replicable in the Bern cohort (*β* = −53.06, uncorrected *p* = .03, *p* > .05, corrected) (see Figure 1). Additionally in the Mannheim cohort only, the right accumbens was nominally significantly reduced (*β* = −31.27, *p* = .018 uncorrected, *p* > .089 corrected), but this did not survive FDR correction.

**Figure 2.**
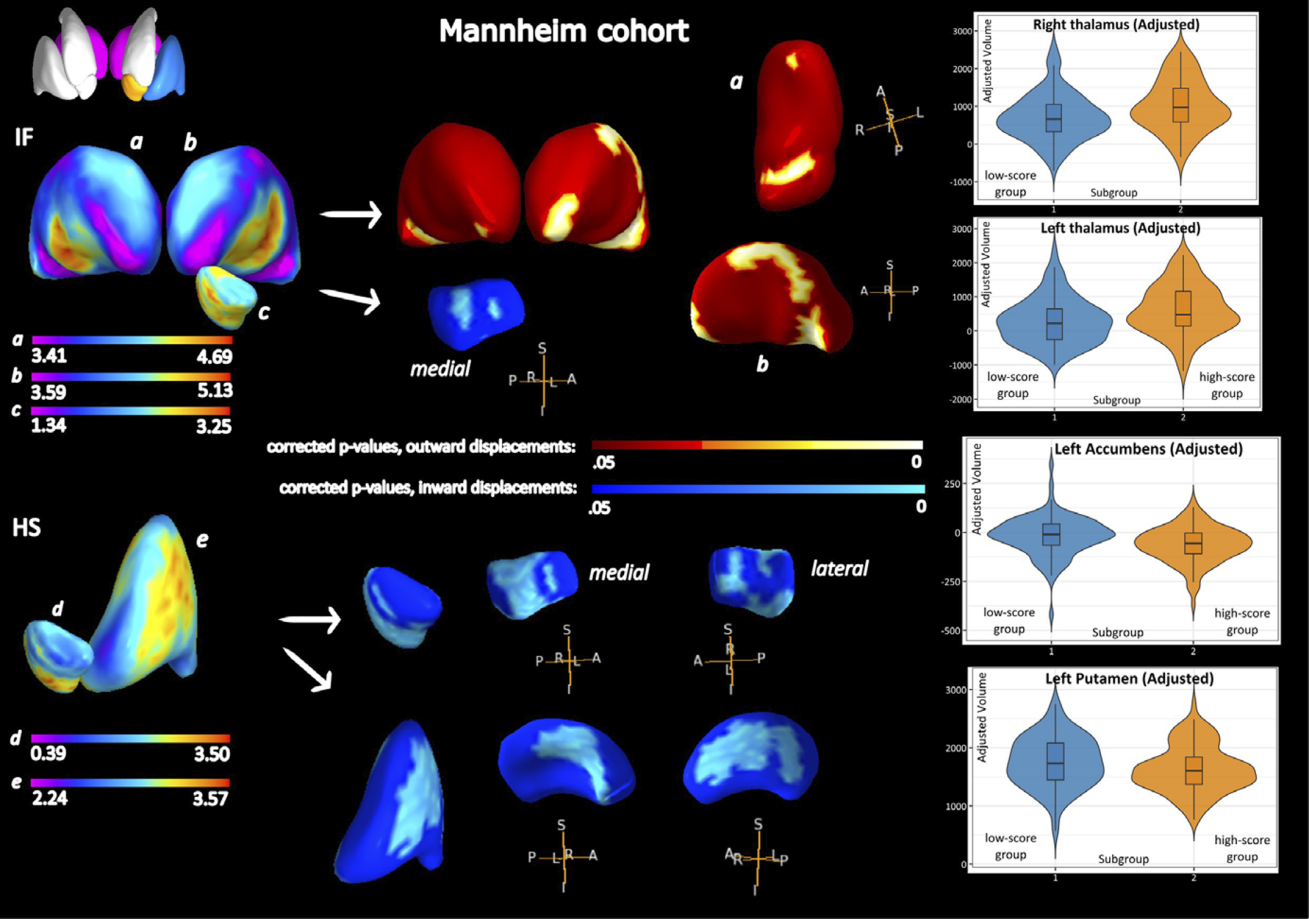
In the Mannheim cohort, integrative function (IF) deficits were associated with smaller accumbens (c) and larger thalamus volumes (a, b), and hard signs (HS) with reduced left putamen (e) and accumbens volumes (d). Left: raw t-maps and corrected-p-maps of surface displacements. Right: adjusted volumes for the subgroups (1: low NSS score, 2: high NSS score). Figure 2. long description.

*MOCO:* No significant group differences in either cohort. *IF:* In Mannheim, higher scores were associated with reduced left accumbens (*β* = −50.57, *p* < .001 uncorrected, *p* = .004 corrected; Figure 2) and increased bilateral thalamic volumes (right: *β* = 310.70, *p* = .001 uncorrected, *p* = .004 corrected; left: *β* = 337.58, *p* = .001 uncorrected, *p* = .004 corrected; Figure 2). Neither of these results were replicable in the Bern cohort; however, a similar trend of mean shifts could be observed (right: *β* = 278.25, left: *β* = 219.47, both *p* > .05 uncorrected and corrected). *COMT:* Reduced left accumbens volume was found in the high-NSS group in Mannheim (*β* = −41.05, *p* = .004 uncorrected, *p* = .037 corrected), which was not replicable in the Bern cohort, where a shift in the same direction could be observed but remained non-significant (*β* = −14.94, *p* = .550 uncorrected, *p* = .749 corrected). *RLSPO:* No significant differences in Mannheim. *HS:* In Mannheim, the high-NSS group exhibited significantly reduced volumes in the left accumbens (*β* = −43.63, *p* = .002 uncorrected, *p* = .017 corrected; Figure 2). No subscale for both, RLSPO and HS, existed in the NES. Similar trends were observed in the right accumbens and left putamen, although these did not survive multiple comparison correction (p = .13).

### Relationship between NSS scores and basal ganglia volumes

Across cohorts, follow-up analyses were restricted to BGT structures that showed significant volumetric differences between the high- and low-NSS groups. Overall, associations between regional volume and clinical or neurological measures were not broadly consistent across cohorts, suggesting that most effects were cohort-specific rather than directly replicable.

**Figure 3.**
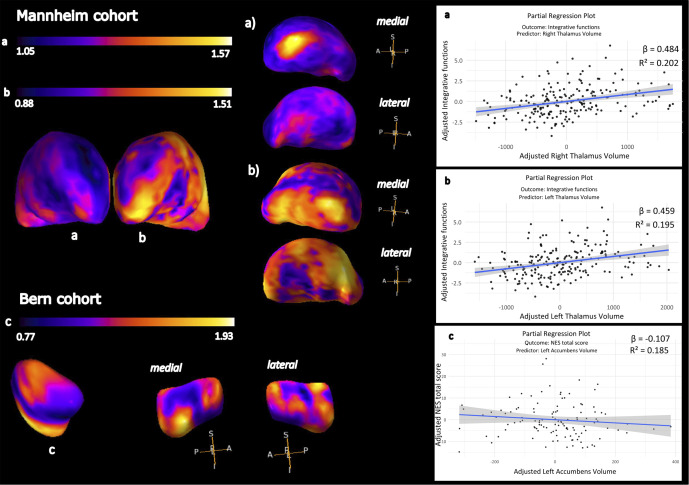
Left side: Raw t-maps of surface displacements associated with IF in the right and left thalamus (a, b) and with NES total score in the left accumbens (c). Right side: partial regression plots of thalamic volumes (a, b) and left accumbens volume (c). Figure 3. long description.

In the Mannheim cohort, significant associations emerged in the low-NSS (*n* = 127) group only. Larger right and left thalamic volumes were associated with higher IF scores (right: *β* = .43, R^2^ = .09, *p* < .05; left: *β* = .39, R^2^ = .08, *p* < .05; Figure 3).This association was not observed in the high-NSS group (n = 81). Surface-based analyses of the thalamus showed predominantly positive correlations between local surface displacement and IF scores. In the right thalamus, outward displacement was localized mainly to medial surfaces corresponding to dorsomedial nuclei, whereas the left thalamus showed a more diffuse pattern involving anterior, ventrolateral, and ventroposterior surfaces, potentially implicating pulvinar and ventrolateral nuclei. However, none of these clusters survived permutation-based FWE correction. Raw t-maps of the thalamus are illustrated in Figure 3.

In the Bern cohort, a different structure showed an association with NSS severity. In the low-NSS subgroup, left accumbens volume was significantly associated with NES total scores (*β* = .32, R^2^ = .10, *p* = .03; Figure 3). Surface-based analysis of the left accumbens revealed a heterogeneous pattern of vertex-wise displacement associated with NSS severity. Although no clusters survived FWE correction, the uncorrected maps indicated localized outward displacement along ventroposterior and medial surfaces (raw t-maps in Figure 3), likely corresponding to the accumbens shell, alongside inward displacement on dorsal and lateral surfaces. Together, these findings indicate that the lack of PANSS associations was replicable across cohorts, whereas the observed structure–NSS relationships were cohort-specific: thalamic associations with IF scores were unique to Mannheim, and left accumbens associations with NES total scores were unique to Bern.

#### Group differences: SSD patients vs healthy controls

Significant differences in NSS high-score and NSS low-score groups compared to the HC in both cohorts are displayed in Table 3. Overall, patients showed higher subcortical volumes than HC in both cohorts, particularly in the nucleus caudatus, putamen, pallidum, and thalamus. However, in the Bern cohort, most mean shifts remained non-significant after FDR correction (Table 3).

**Table 3.** Structures showing significant volumetric differences between SSD patients with NSS *high-score* and healthy controls (HC) as well as SSD patients with NSS *low-score* and HC Table 3. long description.

Cohort	Comparison	NSS score	Basal ganglia – Thalamus	β-value^a^	p-values^b^
Mannheim	*NSS high-score group* vs *HC*	Total score	Right caudate	167.40	*p* = .018* | p(corr) = .060
			Left putamen	208.10	*p* = .004* | p(corr) = .020*
			Right putamen	156.40	*p* = .025* | p(corr) = .064
			Left pallidum	101.90	*p* < .001** | p(corr) = .007*
		MOCO	Left caudate	125.19	*p* = .049* | p(corr) = .082
			Right caudate	193.07	*p* = .005* | p(corr) = .012*
			Left putamen	208.03	*p* = .003* | p(corr) = .011*
			Right putamen	203.66	*p* = .002* | p(corr) = .011*
			Left pallidum	94.29	*p* = .001* | p(corr) = .011*
		IF	Right caudate	173.95	*p* = .026* | p(corr) = .044*
			Left putamen	214.55	*p* = .004* | p(corr) = .010*
			Right putamen	145.56	*p* = .047* | p(corr) = .068*
			Left pallidum	141.86	*p* < .001** | p(corr) < .001**
			Right pallidum	93.93	*p* < .001** | *p*(corr) < .001**
			Left thalamus	373.76	*p* = .003* | p(corr) = .010*
			Right thalamus	402.04	*p* = .001* | p(corr) = .007*
		RLSPO	Right caudate	150.17	*p* = .041* | p(corr) = .069*
			Left putamen	261.06	*p* < .001** | p(corr) = .003*
			Right putamen	194.97	*p* = .006* | p(corr) = .021*
			Left pallidum	104.05	*p* < .001** | p(corr) = .003*
			Right pallidum	80.75	*p* = .016* | p_FDR = .040*
			Right thalamus	256.07	p = .035* | p(corr) = .069*
		COMT	Right caudate	140.15	p = .035* | p(corr) = .071*
			Left putamen	176.79	p = .013* | p(corr) = .052*
			Right putamen	160.80	p = .017* | p(corr) = .052*
			Left pallidum	94.55	p < .001** | p(corr) = 0.002*
			Right pallidum	60.17	*p* = .021* | p(corr) = 0.052*
		HS	Right caudate	173.84	*p* = .011* | p(corr) = 0.039*
			Left putamen	176.90	*p* = .011* | p(corr) = 0.039*
			Right putamen	158.08	*p* = .020* | p(corr) = 0.043*
			Left pallidum	106.66	*p* < .001** | p(corr) = .008*
			Right pallidum	72.51	*p* = .021* | p(corr) = .043*
	*NSS low-score* group vs *HC*	Total score	Right caudate	164.87	*p* = .005* | p(corr) = .011*
			Left putamen	241.71	*p* < .001** | p(corr) = .002*
			Right putamen	241.17	*p* < .001** | p(corr) = .002*
			Left pallidum	96.01	*p* = .001* | p(corr) = .003*
			Right pallidum	77.94	*p* = .002* | p(corr) = .006*
		MOCO	Right caudate	140.62	*p* = .019* | p(corr) = .032*
			Left putamen	259.12	*p* < .001** | p(corr) = .002*
			Right putamen	204.59	*p* = .003*| p(corr) = .008 *
			Left pallidum	108.22	*p* < .001** | p(corr) = .002*
			Right pallidum	87.80	*p* < .001** | p(corr) = .003*
			Left thalamus	240.91	*p* = .03* | p(corr) = .048*
			Right thalamus	262.91	*p* = .016* | p(corr) = .032*
		IF	Right caudate	165.58	*p* = .003* | p(corr) = .011*
			Left putamen	235.90	*p* < .001** | p(corr) = .002*
			Right putamen	238.77	*p* < .001** | p(corr) = .002*
			Left pallidum	75.12	*p* = .004* | p(corr) = .011*
			Right pallidum	61.62	*p* = .022* | p(corr) = .044*
		RLSPO	Right caudate	171.19	*p* = .003* | p(corr) = .010*
			Left putamen	196.76	*p* = .004* | p(corr) = .010*
			Right putamen	203.30	*p* = .002* | p(corr) = .010*
			Left pallidum	94.92	*p* = .001* | p(corr) = .010*
			Right pallidum	66.06	*p* = .011* | p(corr) = .022*
		COMT	Left caudate	132.25	*p* = .017* | p(corr) = .029*
			Right caudate	189.24	*p* = .002* | p(corr) = .006*
			Left putamen	285.20	*p* < .001** | p(corr) < .001**
			Right putamen	253.84	*p* < .001** | p(corr) = .001*
			Left pallidum	106.04	*p* = .001* | p(corr) = .005*
			Right pallidum	91.78	*p* = .004* | p(corr) = .009*
		HS	Right caudate	153.36	*p* = .009* | p(corr) = .019*
			Left putamen	277.39	*p* < .001** | p(corr) = .001*
			Right putamen	246.57	*p* < .001** | p(corr) = .001*
			Left pallidum	87.68	*p* = .001* | p(corr) = .003*
			Right pallidum	68.04	*p* = .006* | p(corr) = .017*
Bern	*NES high-score group* vs *HC*	MOCO	Left caudate	179.51	*p* = .029* | p(corr) = .138
			Right caudate	225.78	*p* = .041* | p(corr) = .138
			Left putamen	379.86	*p* = .013* | p(corr) = .134
		IF	Right caudate	210.17	*p* = .022* | p(corr) = .167
			Left putamen	268.84	*p* = .047* | p(corr) = .167
			Right putamen	232.37	*p* = .05 | p(corr) = .167
	*NES low-score group* vs *HC*	Total score	Left caudate	265.42	*p* = .006* | p(corr) = .047 *
			Right caudate	285.56	*p* = .030* | p(corr) = .101
			Left putamen	478.41	*p* = .009* | p(corr) = .047 *
		IF	Left caudate	254.18	*p* = .012* | p(corr) = .062
			Left putamen	477.84	*p* = .012* | p(corr) = .062
		COMT	Left caudate	249.04	*p* = .011* | p(corr) = .067
			Right caudate	266.32	*p* = .048* | p(corr) = .162
			Left putamen	465.43	*p* = .013* | p(corr) = .067

*Note:* NSS, Heidelberg Neurological Soft Signs Scale; NES, Neurological Evaluation Scale; MOCO, motor coordination; IF, integrative function; RLSPO, right/left and spatial orientation; COMT, complex motor tasks; HS, hard signs; HC, healthy controls.

a Positive values indicate higher volumes for patients vs controls, negative values smaller volumes for patients vs controls.

b uncorrected | corrected, with *p < .05, ^**^p < .001.

#### Daily antipsychotic medication and duration of illness mediating/moderating the NSS-basal ganglia-thalamic relationship

Daily antipsychotic medication and DOI did not explain or modify the observed structure–symptom associations in either cohort. In the Mannheim cohort, the association between left and right thalamic volume and IF in the low-score group was not mediated by medication or DOI (mediation effects *p* > .05, Figure 4.1). No moderation effects could be detected for these two variables as well (interaction terms *p* > .05, Figure 4.1). In the Bern cohort as well, both medication and DOI showed no significant mediation or moderation effects on the left accumbens volume-NES total score association previously found in the low-score group (*p* > .05, Figure 4.2).

**Figure 4.**
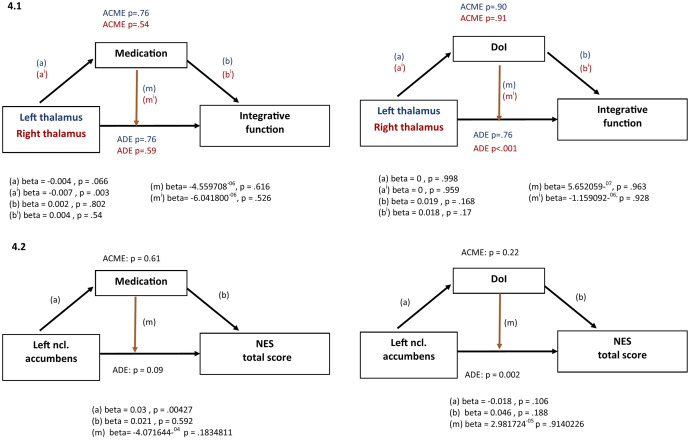
Mediation and moderation effects of medication (left) and duration of illness (DOI, right) in the Mannheim cohort (4.1) and in the Bern cohort (4.2). Figure 4. long description.

## Discussion

This bicentric MRI study investigated BGT correlates of NSS in SSD in one of the largest samples examined to date, combining structural data from two independent patient cohorts. The findings can be summarized around three main observations: First, NSS severity was associated with reduced left nucleus accumbens volume, with a corrected significant effect in the Mannheim cohort and an uncorrected effect in the same direction in the Bern cohort. Second, additional NSS subdomain-related effects involved the nucleus accumbens, putamen, and thalamus, particularly for IF and HS, but these findings were more cohort-specific. Third, associations between BGT morphology and NSS severity were evident primarily in patients with lower NSS burden, with no corresponding effects in high-NSS subgroups. Surface-based analyses revealed exploratory thalamic shape alterations related to IF, particularly in anterior and dorsomedial regions, with more pronounced alterations in anterior regions of the left thalamus. Daily antipsychotic medication and DOI did not mediate or moderate the tested NSS–BGT associations in either cohort. Together, these results provide partial cross-cohort convergence for nucleus accumbens involvement in NSS, while thalamic and surface-based findings appear more cohort-specific and should be interpreted cautiously.

NSS can be understood within a neurodevelopmental framework, as converging evidence indicates that they reflect early deviations in brain maturation across different populations. Early neuroimaging work by Dazzan et al. (2004) and Morgan et al. (2006) showed that higher NSS are associated with structural alterations in cortical and subcortical regions – including BGT – in both first-episode psychosis and healthy individuals, suggesting that NSS index trait-like neurobiological variation rather than illness-related effects alone. This view was further supported by studies from Gay et al. (2013, 2017), demonstrating that patients with elevated NSS exhibit altered cortical folding patterns and that NSS interact with other neurodevelopmental markers (e.g. anterior cingulate cortex morphology, ventricular enlargement) to influence cognitive control. Longitudinal and high-risk studies extend these findings: work by Dean and Mittal (2015), Mittal et al. (2010) and Mittal et al. (2013) shows that motor abnormalities in adolescents at clinical high risk predict later conversion to psychosis and are linked to striatal alterations, while Burton et al. (2023) demonstrated persistent motor coordination deficits in children at familial risk that are associated with later psychotic experiences. More recent evidence further indicates that NSS and related neurodevelopmental markers are elevated in patients and, to a lesser degree, in unaffected relatives, supporting a continuum of vulnerability (Giralt-Lopez et al., 2025). Taken together, these findings across healthy individuals, at-risk populations, and patients suggest that NSS represent a transdiagnostic marker of aberrant neurodevelopment, likely reflecting early disruptions in cortico-striato-thalamo-cortical circuits. In this context, our findings are consistent with a neurodevelopmental interpretation of NSS, although the cross-sectional design of the present study does not allow direct conclusions about developmental trajectories.

The reduction of left nucleus accumbens volume in patients with higher NSS burden represents the most consistent BGT finding of the present study. This result supports and extends previous neuroimaging research, highlighting a central role of BGT structures in the neurobiology of NSS in SSD. Specifically, our results converge with earlier MRI studies linking greater NSS burden to structural alterations in subcortical regions such as BG and brainstem, particularly in early or first-episode SSD (Dazzan et al., 2004; Hirjak, Wolf, Schröder, & Thomann, 2012, 2013; Venkatasubramanian et al., 2003; Venkatasubramanian, Jayakumar, Gangadhar, & Keshavan, 2008). Our findings also align with Quispe Escudero and Schröder (2020), who demonstrated that increased motor NSS scores correlate with reduced grey matter volume (GMV) in frontal, cerebellar, and subcortical regions, especially the thalamus and caudate nucleus, using voxel-based morphometry. Similarly, Hirjak et al. (2012), using the same segmentation method as in our study (FSL-FIRST), reported regionally specific shape alterations in the thalamus, caudate nucleus, and globus pallidus associated with NSS in recent-onset SSD. By identifying the left nucleus accumbens as the most consistent cross-cohort structure, our data add evidence for ventral striatal involvement in NSS and suggest that NSS-related BGT alterations may involve not only dorsal motor circuits but also regions implicated in limbic, motivational, and sensorimotor integration.

The observed left-lateralized predominance aligns with neurobiological models that highlight atypical hemispheric asymmetry as a feature of SSD (Okada et al., 2016; Schijven et al., 2023). The left hemisphere plays a central role for the planning and sequencing of complex motor acts. Structural deficits in these left-sided structures may, therefore, be involved in the neurobiology of NSS. However, given that only the left nucleus accumbens finding showed partial cross-cohort convergence, this lateralization should be interpreted cautiously. Our findings are compatible with a localized contribution of left-hemisphere cortico-striatal and sensorimotor circuits to NSS in SSD, rather than indicating a generalized bilateral subcortical decline. Taken together, these findings consistently implicate a network of subcortical structures including thalamus, striatum, and ventral BG in the pathophysiology of NSS. Our study extends this literature by demonstrating that volume reductions in these regions are most pronounced in SSD patients with more severe NSS across two independent cohorts, supporting the notion that NSS represent a stable, trait-like marker of subcortical dysfunction in SSD. The present study extends this literature by showing that volume reductions were most consistent for the left nucleus accumbens in patients with higher NSS burden, while other BGT findings were more cohort-specific. Thus, NSS may represent markers of subcortical involvement in SSD, but the current results support partial rather than uniform reproducibility across BGT regions.

In particular, vertex-wise analysis suggested localized deformation of the medial nucleus accumbens, potentially corresponding to the nucleus accumbens shell. This region receives ventromedial prefrontal inputs and is implicated in reward and motivation, whereas the core is more closely linked to dorsal prefrontal input, conditioned responses, and goal-directed behavior (Baliki et al., 2013; Salgado & Kaplitt, 2015). T-maps indicated outward displacement in posterior shell regions and inward displacement medially, possibly reflecting altered limbic–prefrontal balance within the nucleus accumbens (Marinescu & Labouesse, 2024). Such imbalance may affect motor gating and the integration of emotional and sensory information into coordinated action, contributing to NSS.

Thalamic findings were most evident for IF and were more cohort-specific than the nucleus accumbens volume effect. Notably, the NSS–BG association was absent in the high-NSS subgroup, suggesting potential non-linear or stage-dependent effects in thalamic-NSS relationships. This pattern echoes the synthesis by Samson et al. (2022), who identified the thalamus as a central node in the neurobiology of NSS, particularly in relation to deficits in IF and COMT. Several earlier studies corroborated this link (Thomann, Bachmann, & Schröder, 2009), and Hirjak et al. (2012) reported significant associations between higher NSS and thalamic atrophy in first- and recent-onset SSD, with specific relevance to integrative and motor domains. Quispe Escudero and Schröder (2020) also found that motor NSS subscores were negatively correlated with GMV in the thalamus and related regions in first-episode psychosis. The absence of significant thalamic-NSS correlations in the high-NSS subgroup in our study may reflect a ceiling effect, structural saturation, or reduced variability in late-stage or more severely affected patients, whereas low-NSS individuals may retain variability that still maps onto brain structure. These findings, therefore, suggest that BGT–NSS relationships may not be strictly linear across the full NSS severity range.

In contrast to the observed thalamic effects, left nucleus accumbens volume did not show significant associations with global NSS severity or any NSS subdomains in the Mannheim cohort, neither in high- nor low-NSS groups. This lack of association may appear inconsistent with the group-level differences detected between high- and low-NSS subgroups, but it may reflect restricted variance within subgroups or non-linear associations that defy simple linear modeling (Iacobucci et al., 2015; Rucker, McShane, & Preacher, 2015). Importantly, Samson et al. (2022) highlighted that only a minority of studies have systematically examined the nucleus accumbens in relation to NSS, and where reported, results were heterogeneous. This discrepancy in the literature – and in our findings – may arise from the structural and functional heterogeneity of the nucleus accumbens, which comprises core and shell subdivisions with distinct connectivity profiles and functional implications for sensorimotor and limbic integration (Groenewegen & Trimble, 2007; Meredith, Baldo, Andrezjewski, & Kelley, 2008). Given the role of the ventral striatum in motivational and reward-based processes, the functional relevance of nucleus accumbens alterations for MOCO or IF may be less direct compared to other BG structures. Thus, the nucleus accumbens finding may be more apparent at the group-comparison level than in linear within-subgroup associations.

Thalamic surface analyses suggested topographically specific alterations associated with IF, although these effects did not survive TFCE correction and should, therefore, be interpreted cautiously. Raw t-maps indicated outward displacement in regions corresponding to dorsomedial, ventroanterior, ventroposterior, and pulvinar nuclei of the left thalamus. These nuclei are involved in motor, sensory, attentional, and associative processing: anterior ventral nuclei are closely connected to motor circuits, ventroposterior nuclei relay sensory information, and the pulvinar supports multimodal sensory processing, visual attention, and salience (Marcuse, Langan, & Fields, 2025). The dorsomedial nucleus connects with prefrontal, cingulate, insular, brainstem, and limbic regions, supporting executive and emotional integration (Marcuse, Langan, & Fields, 2025; Mitchell, 2015; Parnaudeau, Bolkan, & Kellendonk, 2018). It acts as an integrative hub, facilitating reciprocal communication between the prefrontal cortex and limbic structures to support executive functions and emotional regulation. Overall, these regions form part of cortico-cerebellar and frontoparietal circuits relevant to sensorimotor integration and attention (Samson, Lahti, & Kraguljac, 2022).

The functional relevance of thalamic alterations for NSS may be understood in the context of its central role within cortico-striato-thalamo-cortical circuits. The thalamus acts as a key relay integrating motor, sensory, and associative information, thereby supporting sensorimotor coordination and higher-order integration processes that are impaired in NSS (Janssen et al., 2009). While most neuroimaging studies on NSS across healthy individuals, at-risk populations, and SSD patients have relied on structural MRI, emerging functional evidence provides complementary insight. For example, an earlier task-based fMRI study in healthy individuals have shown that increasing motor sequencing complexity is accompanied by enhanced thalamic activation alongside sensorimotor and cerebellar regions, underscoring its role in motor integration (Chan et al., 2006). In contrast, one resting-state fMRI study suggested that NSS in healthy subjects are primarily related to cortical network activity, with limited thalamic involvement, indicating that subcortical contributions may become more prominent under pathological conditions (Thomann et al., 2015). Together, these findings support the interpretation that thalamic alterations in SSD reflect disrupted integration within distributed sensorimotor networks underlying NSS. Our findings could reflect a compensatory expansion or a failure of normal pruning within critical sensory-relay and executive hubs, suggesting that structural remodeling in these regions is a key correlate of impaired multimodal synthesis in SSD.

Daily antipsychotic medication, DOI, and general clinical symptom severity did not explain the tested NSS–BGT associations. Further, the specific NSS-related BG alterations did not correlate with positive or negative symptom severity. This is important because antipsychotics are known to influence subcortical morphology, particularly striatal volumes (Yang & Lui, 2021). However, several clinical studies indicated that NSS are relatively independent of antipsychotic medication effects. While literature suggested that NSS severity can respond to antipsychotic treatment alongside symptom remission (Bachmann & Schröder, 2018; Mayoral, Bombín, & Arango, 2012), NSS manifestation itself remains independent of daily dosage (Fritze et al., 2020; Petrescu & Ciobanu, 2022) and is also prevalent in medication-naïve SSD patients. In ultra-high-risk adolescents, (Pitzianti et al., 2019) found no differences in NSS between antipsychotic-treated and drug-naïve individuals. Similarly, Fritze et al. (2020) showed that daily antipsychotic dosage did not significantly affect NSS severity in SSD, supporting a genuine rather than medication-induced origin of NSS. Petrescu and Ciobanu (2022) likewise reported that chlorpromazine-equivalent dose was not a significant determinant of NSS in schizophrenia patients with predominant negative symptoms. Longitudinal data further suggested that NSS remain relatively stable over time and do not simply improve in parallel with clinical symptom change or antipsychotic response (Fountoulakis et al., 2019). Although medication is known to influence BG morphology in SSD (Yang & Lui, 2021), our results align with Dazzan et al. (2004) in identifying medication-independent BG alterations associated with NSS. At the same time, NSS have been linked to treatment-resistant and neurodevelopmentally loaded subgroups of SSD (Iasevoli et al., 2018; Vellucci et al., 2025), suggesting that they may index a more trait-like dimension of illness biology. In summary, our findings support the conclusion that the BG are fundamentally involved in NSS pathophysiology, reflecting sensorimotor circuit pathology that is largely separable from daily antipsychotic medication exposure, DOI, and positive, negative and general symptom severity.

Although the present study focused on BGT morphology, NSS are unlikely to arise from isolated BGT abnormalities alone. Together, the data from both the Mannheim and Bern cohorts highlight the central role of BGT structures, particularly the thalamus and striatum, including the nucleus accumbens and putamen, in the neurobiology of NSS in SSD. These findings are in line with the synthesis by Samson et al. (2022), who identified BG and cerebellar structures as among the most consistent anatomical correlates of NSS across structural MRI studies. Extending this structural perspective, early and subsequent fMRI studies have demonstrated that NSS-related behaviors engage distributed cortico-subcortical circuits, including sensorimotor cortex, BGT, and cerebellum. For example, reduced activation of sensorimotor and supplementary motor areas in schizophrenia (Schroder et al., 1995; Schroder et al., 1999) altered BG and cerebellar engagement during motor tasks (Muller, Roder, Schuierer, & Klein, 2002), and task-dependent recruitment of thalamic regions in motor sequencing paradigms (Chan et al., 2006) all point toward dysfunction within integrated motor networks. Meta-analytic evidence further supported a cerebello-thalamo-prefrontal network model of NSS (Zhao et al., 2014), while more recent studies suggested aberrant frontoparietal connectivity and compensatory activation patterns during complex motor processing (Zemankova et al., 2016). Importantly, findings in healthy individuals indicated that NSS variability may predominantly reflect cortical network efficiency under physiological conditions (Hirjak et al., 2016; Thomann et al., 2015), whereas subcortical contributions – including thalamic and striatal dysfunction – may become more pronounced in clinical populations. In this context, our use of surface-based morphometry provides a more fine-grained characterization of BGT deformation patterns, which may help reconcile inconsistencies in prior volumetric studies that did not account for intra-structural heterogeneity. Finally, NSS are not only linked to GMV alterations but have also been associated with abnormalities in major white matter tracts, both in SSD patients (e.g. Viher et al., 2022) and in healthy individuals (Hirjak et al., 2017), suggesting that disrupted structural and functional connectivity within distributed motor and integrative networks may represent a key mechanism underlying NSS.

### Strengths and limitations

A major strength of this study is the exploration of BGT structures and NSS in two independent cohorts of SSD patients. We compared these two cohorts separately with two samples of matched HC. Moreover, we analyzed two independent SSD patient cohorts in which NSS were assessed using different clinical instruments. Although these scales differ in their structure, they assess partially overlapping NSS items. Despite these methodological differences, the results showed a consistent pattern across the two cohorts, suggesting that the observed associations are unlikely to be driven by a specific measurement approach. Nevertheless, several important limitations should be considered. First, no inference on the pathophysiology of NSS in SSD can be made due to the cross-sectional design of the study. Moreover, some findings did not remain significant after correction for multiple comparisons, which was expected given that multiple BGT structures and NSS subscales were examined; these null results may also partly reflect limited statistical power. Second, environmental factors may also influence NSS expression in SSD and should be considered when interpreting NSS as markers of neurodevelopmental vulnerability. Early studies linked NSS to obstetric complications, although findings were inconsistent: Lane et al. (1996) reported higher NSS in male patients whose mothers had obstetric complications, whereas Peralta et al. (2006) found associations between obstetric complications and neurological abnormalities in neuroleptic-naïve psychotic patients, while Boks et al. (2007)reported the opposite pattern. Cannabis use represents another relevant environmental factor. Dervaux et al. (2013) found increased NSS in cannabis dependence, whereas Ruiz-Veguilla et al. (2009), Løberg et al. (2014), Mhalla et al. (2017), and Mallet et al. (2017) suggested that cannabis-associated psychosis may show fewer NSS, possibly reflecting lower neurodevelopmental vulnerability. More recent work showed NSS and altered sensorimotor circuits in heavy cannabis users (Wolf et al., 2021; Wolf et al., 2023). However, we found no studies on the influence of urban upbringing and migration on NSS. Still, NSS likely reflect interactions between early neurodevelopmental liability and later environmental exposures. Lastly, since the NSS and NES are motor scales, handedness could play a role in the association with subcortical regions, which was due to missing values not adjusted for.

## Conclusion

In conclusion, this bicentric study provides partial cross-cohort evidence that NSS severity in SSD is associated with BGT morphology, with the strongest convergence observed for reduced left nucleus accumbens volume. Thalamic and subdomain-specific effects, particularly those related to IF, were biologically plausible but more cohort-specific and exploratory. These findings support a role of ventral striatal and thalamic circuits in NSS while emphasizing that NSS likely reflect dysfunction across distributed sensorimotor and integrative networks.

## Data Availability

The data that support the findings of this study are available on reasonable request from the respective studies. Data-request applications will be approved by the committees of the studies.

## Long descriptions

### Table 1. Long description

The table is divided into two main columns for the Mannheim cohort and the Bern cohort, each further subdivided into S S D and H C groups.

1. Mannheim Cohort:

* S S D (n = 209): Mean age 38.14 (S D 12.01), range 18 to 65. Sex is 124 male (59 percent). Right-handedness is 93.3 percent.

* H C (n = 75): Mean age 36.75 (S D 11.65), range 18 to 58. Sex is 43 male (57 percent). Handedness is not available.

* Statistical significance for age between these groups is p = .39.

2. Bern Cohort:

* S S D (n = 118): Mean age 36.61 (S D 12.14), range 18 to 60. Sex is 62 male (52.54 percent). Handedness is not available.

* H C (n = 59): Mean age 37.41 (S D 12.44), range 20 to 59. Sex is 31 male (52.54 percent). Handedness is not available.

* Statistical significance for age between these groups is p = .74.

3. Cross-Cohort Comparisons:

* Mean age difference between patient samples: p = .29.

* Mean age difference between control samples: p = .75.

* Overall sex distribution significance: p = .23.

Navigate back to Table 1..

### Table 2. Long description

The table presents data for 327 patients across two cohorts: Mannheim (n = 209) and Bern (n = 118).

Key clinical characteristics include:

* N S S total score: Mannheim mean 19.57 (S D 8.81); Bern not available.

* N E S total score: Mannheim not available; Bern mean 13.69 (S D 9.37).

* P A N S S total score: Mannheim 66.8 (S D 19.26) versus Bern 70.69 (S D 18.56), p = .08.

* P A N S S negative symptoms: Mannheim 17.18 (S D 7.42) versus Bern 20.03 (S D 6.92), p < .001.

* P A N S S positive symptoms: Mannheim 15.53 (S D 6.29) versus Bern 14.64 (S D 4.92), p = .19.

* Duration of illness (D O I): Mannheim 10.8 years versus Bern 10.63 years, p = .89.

* O L Z equivalent: Mannheim 16.39 mg/day versus Bern 14.02 mg/day, p = .13.

N S S and N E S subscales (mean, S D, median):

* Mannheim Motor coordination: 7.50 (4.15), 7.

* Bern Motor coordination: 2.06 (2.23), 1.

* Mannheim Integrative functions: 3.42 (1.91), 3.

* Bern Integrative functions: 2.54 (2.54), 2.

* Mannheim Complex motor tasks: 3.41 (2.17), 3.

* Bern Sequencing: 4.23 (3.34), 4.

Medication status:

* Mannheim: 195 medicated, 14 unmedicated.

* Bern: 107 medicated, 5 unmedicated, p = .42.

Movement and Catatonia scales:

* S A S: Mannheim 3.44 (S D 2.95).

* A I M S: Mannheim 1.07 (S D 2.47) versus Bern 0.92 (S D 2.82), p = .617.

* N C R S: Mannheim 4.23 (S D 4.19).

* B F C R S: Bern 3.83 (S D 4.14).

Navigate back to Table 2..

### Figure 1. Long description

The display is divided into two horizontal sections for the Mannheim cohort and Bern cohort.

Mannheim cohort section:

* Left: A three-dimensional anatomical model of the nucleus accumbens. A heat map on the surface shows t-values ranging from 0 (purple) to 3.79 (yellow). Arrows point to isolated medial and lateral views of the structure showing corrected p-values. A color bar at the top indicates corrected p-values from .05 (dark blue) to 0 (light cyan).

* Right: A violin plot titled Left Accumbens (Adjusted). The y-axis represents Adjusted Volume from -500 to 250. Subgroup 1 (low-score group) in blue shows a slightly higher median and wider distribution than Subgroup 2 (high-score group) in orange.

Bern cohort section:

* Left: Similar three-dimensional anatomical model. The t-map scale ranges from 0.01 to 3.48. A single arrow points to a medial view showing corrected p-values. An anatomical axis key indicates Superior (S), Inferior (I), Posterior (P), and Anterior (A) directions.

* Right: A violin plot titled Left Accumbens (Adjusted). The y-axis represents Adjusted Volume from 0 to 750. Subgroup 1 (low-score group) in blue shows a higher median volume compared to Subgroup 2 (high-score group) in orange.

Navigate back to Figure 1..

### Figure 2. Long description

A multi-panel display divided into two main horizontal sections labeled I F and H S.

Top section I F (Integrative Function):

* Left: Three-dimensional t-maps of the thalamus (labeled a and b) and accumbens (labeled c) showing surface displacements in a blue-to-yellow gradient. Color bars indicate t-values: a (3.41 to 4.69), b (3.59 to 5.13), and c (1.34 to 3.25).

* Middle: Corrected p-value maps showing outward displacements in red-to-yellow (0.05 to 0) and inward displacements in blue-to-cyan (0.05 to 0). Anatomical axes indicate Superior, Inferior, Anterior, Posterior, Left, and Right orientations.

* Right: Two violin plots for Right Thalamus and Left Thalamus. Each plot compares subgroup 1 (low N S S score, blue) and subgroup 2 (high N S S score, orange). Subgroup 2 shows higher adjusted volumes in both thalamic regions.

Bottom section H S (Hard Signs):

* Left: Surface maps for the accumbens (labeled d) and left putamen (labeled e). Color bars indicate t-values: d (0.39 to 3.50) and e (2.24 to 3.57).

* Middle: Medial and lateral views of corrected p-value maps for these structures, predominantly showing blue-shaded inward displacements.

* Right: Two violin plots for Left Accumbens and Left Putamen. Subgroup 2 (high N S S score, orange) shows lower adjusted volumes compared to subgroup 1 (low N S S score, blue) for both the accumbens and putamen.

Navigate back to Figure 2..

### Figure 3. Long description

The figure is divided into three main rows labeled a, b, and c.

Row a (Mannheim cohort):

- Left: A color scale from 1.05 to 1.57 (purple to yellow) above two brain surface maps showing medial and lateral views of the right thalamus. A yellow-hot spot is visible on the medial surface.

- Right: A scatter plot with a positive linear regression line. X-axis is Adjusted Right Thalamus Volume; Y-axis is Adjusted Integrative functions. Statistics: beta = 0.484, R super 2 = 0.202.

Row b (Mannheim cohort):

- Left: A color scale from 0.88 to 1.51 above medial and lateral views of the left thalamus.

- Right: A scatter plot with a positive linear regression line. X-axis is Adjusted Left Thalamus Volume; Y-axis is Adjusted Integrative functions. Statistics: beta = 0.459, R super 2 = 0.195.

Row c (Bern cohort):

- Left: A color scale from 0.77 to 1.93 above three views of the left accumbens.

- Right: A scatter plot with a slight negative linear regression line. X-axis is Adjusted Left Accumbens Volume; Y-axis is Adjusted N E S total score. Statistics: beta = minus 0.107, R super 2 = 0.185.

Spatial orientation markers (S, I, A, P for Superior, Inferior, Anterior, Posterior) accompany each brain map.

Navigate back to Figure 3..

### Table 3. Long description

The table consists of six columns: Cohort, Comparison, N S S score, Basal ganglia – Thalamus, beta-value, and p-values.

In the Mannheim cohort, comparisons between N S S high-score groups and healthy controls show significant positive beta-values across multiple structures including the Right caudate, Left putamen, Right putamen, and Left pallidum for Total score, M O C O, I F, R L S P O, C O M T, and H S sub-scores. Notable values include a beta-value of 402.04 for the Right thalamus in the I F sub-score group with a corrected p-value of .007.

For the Mannheim N S S low-score group versus healthy controls, similar positive volumetric differences are noted in the Right caudate, bilateral putamen, and bilateral pallidum across all sub-scores, with beta-values ranging from 61.62 to 285.20.

In the Bern cohort, the N E S high-score group versus healthy controls shows significant uncorrected p-values for the Left caudate, Right caudate, and Left putamen in M O C O and I F categories, though corrected p-values are non-significant. The Bern N E S low-score group shows significant corrected p-values for the Left caudate (beta 265.42, p corrected .047) and Left putamen (beta 478.41, p corrected .047) in the Total score category.

Footnotes indicate that positive beta-values signify higher volumes in patients compared to controls, and asterisks denote statistical significance levels at p less than .05 and p less than .001.

Navigate back to Table 3..

### Figure 4. Long description

The figure contains four mediation models arranged in a two by two grid.

Panel 4.1 Top-Left: Mediation by Medication. The independent variables are Left thalamus (blue) and Right thalamus (red). Arrows point to the mediator Medication and the outcome Integrative function. Path a beta is -0.004 for left and -0.007 for right. Path b beta is 0.002 for left and 0.004 for right. Path m beta is -4.559708 super -06 for left and -6.041800 super -06 for right. A C M E p-values are .76 and .54. A D E p-values are .76 and .59.

Panel 4.1 Top-Right: Mediation by D O I. Independent variables are Left and Right thalamus. Path a beta is 0 for both. Path b beta is 0.019 for left and 0.018 for right. Path m beta is 5.652059 super -07 for left and -1.159092 super -06 for right. A C M E p-values are .90 and .91. A D E p-values are .76 and less than .001.

Panel 4.2 Bottom-Left: Mediation by Medication. Independent variable is Left ncl. accumbens. Outcome is N E S total score. Path a beta is 0.03, p = .00427. Path b beta is 0.021, p = 0.592. Path m beta is -4.071644 super -04, p = .1834811. A C M E p is 0.61 and A D E p is 0.09.

Panel 4.2 Bottom-Right: Mediation by D O I. Independent variable is Left ncl. accumbens. Outcome is N E S total score. Path a beta is -0.018, p = .106. Path b beta is 0.046, p = .188. Path m beta is 2.981724 super -05, p = .9140226. A C M E p is 0.22 and A D E p is 0.002.

Navigate back to Figure 4..
