# Replicable subcortical alterations linked to neurological soft signs in schizophrenia spectrum disorders – CORRIGENDUM

**DOI:** 10.1017/S003329172610542X

**Published:** 2026-07-31

**Authors:** Amanda Bellanti, Sebastian Volkmer, Dilsa Cemre Akkoc Altinok, Stefan Fritze, Geva A. Brandt, Heike Tost, Urs Braun, Sofie von Känel, Anastasia Pavlidou, Stephanie Lefebvre, Andreas Meyer-Lindenberg, Sebastian Walther, Dusan Hirjak

The authors regret the inclusion of an error in the above article.

The name of the second author was inadvertently given as Seabastian Volkmer. The correct author name is Sebastian Volkmer.

The article has been corrected.
